# Supplementary material for: Cavβ1 regulates T cell expansion and apoptosis independently of voltage-gated Ca2+ channel function
Source: Nat Commun. 2022 Apr 19;13:2033. doi: 10.1038/s41467-022-29725-3 (PMC9018955; doi:10.1038/s41467-022-29725-3)
Supplement: Supplementary file 1 — Supplementary Information [file 41467_2022_29725_MOESM1_ESM.pdf]

SUPPLEMENTARY INFORMATION

Cavβ1 regulates T cell expansion and apoptosis independently of voltage-gated Ca^{2+} channel function

Serap Erdogmus[1*], Axel R. Concepcion[1*], Megumi Yamashita[2], Ikjot Sidhu[1], Anthony Y. Tao[1], Wenyi Li[1], Pedro P. Rocha[3,4], Bonnie Huang[5,6], Ralph Garippa[7], Boram Lee[8], Amy Lee[9], Johannes W. Hell[8], Richard S. Lewis[10], Murali Prakriya[2], Stefan Feske[1]

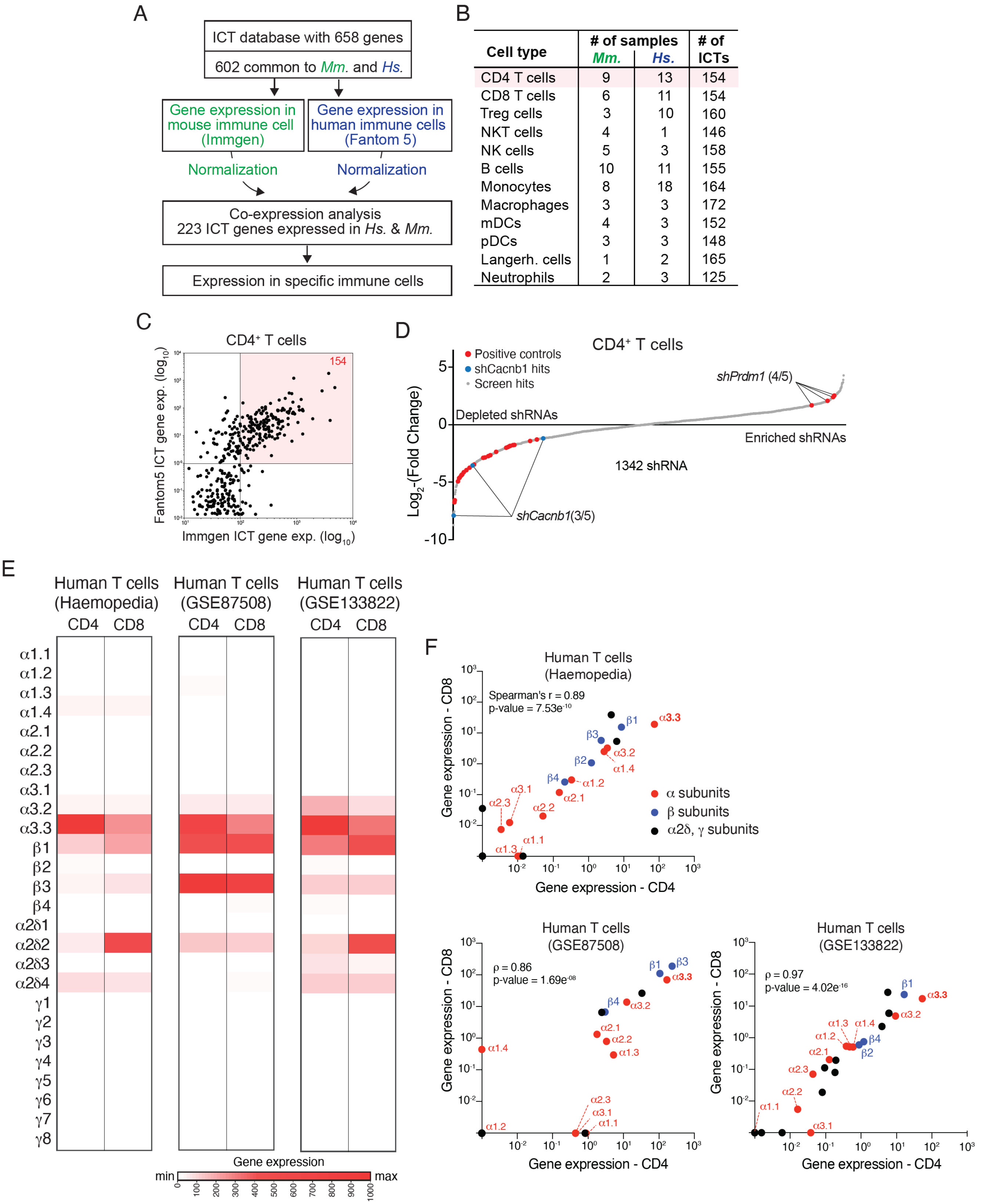

Cell type	# of samples *Mm.*	# of samples *Hs.*	# of ICTs
CD4 T cells	9	13	154
CD8 T cells	6	11	154
Treg cells	3	10	160
NKT cells	4	1	146
NK cells	5	3	158
B cells	10	11	155
Monocytes	8	18	164
Macrophages	3	3	172
mDCs	4	3	152
pDCs	3	3	148
Langerh. cells	1	2	165
Neutrophils	2	3	125

Supplementary Figure 1. Ion channel and transporter (ICTs) expression in immune cells, shRNA screen and comparison of VGCC expression in $CD4^+$ and $CD8^+$ T cells. (A-C) Determination of ICT expression in immune cells. **(A)** Algorithm for ICT database generation and analysis. Of 658 ICTs in our custom ICT library, 602 are annotated in human and mouse genomes. **(B)** Immune cells with expression data available in both ImmGen (mouse) and Fantom5 (human) databases were divided into 12 groups. Listed are numbers of samples per group and numbers of ICT genes expressed > 2-fold above the average of all immune cell subsets. 223 ICT genes were expressed in one or more of the 12 human or mouse immune cell subsets. **(C)** mRNA expression of 223 ICTs in human and mouse $CD4^+$ T cells; 154 ICTs were highly expressed in both species. **(D)** Results of pooled shRNA screen to identify ICTs regulating antiviral immunity by $CD4^+$ T cells. The sigmoidal curve shows the Log_2-fold change depletion (left) or enrichment (right) of cells transduced with 1,342 shRNAs targeting 223 ICTs and positive and negative controls. $CD4^+$ T cells from SMARTA mice that had been transduced with the shRNA library were injected into host mice followed by $LCMV^{ARM}$ infection. At day 7 post-infection, donor $CD4^+$ T cells were analyzed for shRNA depletion by NGS. Individual shRNAs targeting *Cacnb1* are annotated in blue, positive controls in red, and other ICTs in grey. shRNA targeting the transcription factor Blimp-1 (*Prdm1*) served as a positive control. **(E, F)** Correlation of VGCC subunit expression in human $CD4^+$ and $CD8^+$ T cells. **(E)** Heatmaps showing absolute mRNA expression of $\alpha1$, β, $\alpha2\delta$, and γ VGCC subunits in human $CD4^+$ and $CD8^+$ T cells. Expression data was extracted from three independent RNA-Seq datasets: Human Haemopedia, GSE87508, and GSE133822 (Supplementary Table 2). Expression values were rescaled to arbitrary units ranging from 0 to 1000. **(F)** Correlation analysis of mRNA expression for the different VGCC subunits in $CD4^+$ and $CD8^+$ T cells from the 3 RNA-Seq datasets shown in (E). Gene expression values were transformed to gene expression + 0.001 to plot values in log scale. Colored dots represent genes encoding $\alpha1$ (red), β (blue) and $\alpha2\delta$ and γ (black) subunits. Spearman's coefficient (rho) and *p*-values were used to measure the strength of the linear correlation between 2 datasets.

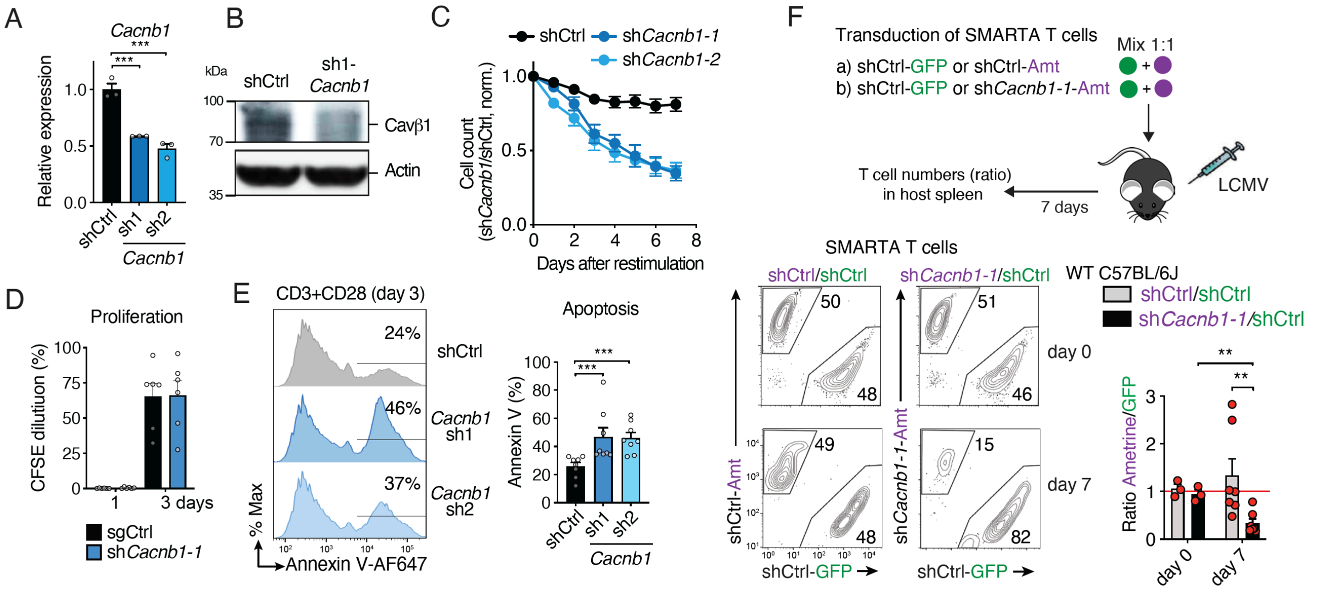

Supplementary Figure 2. Deletion of Cavβ1 impairs viability of CD4+ T cells and their expansion after viral infection. (A) mRNA expression of *Cacnb1* in CD4+ T cells transduced with control shRNA (shCtrl) and 2 individual shRNA targeting *Cacnb1*. mRNA levels were measured in sorted transduced (Amt+) T cells 3 days after transduction and quantified by qPCR. *Rlp32* mRNA was used as housekeeping control, and relative *Cacnb1* levels were normalized to shCtrl. Data are the mean ± SEM of n=3 mice from independent experiments. **(B)** Representative Western blot of Cavβ1 protein in CD4+ T cells transduced with *Cacnb1*-specific or control shRNAs. After 4-5 days, Cavβ1 was detected using a monoclonal antibody recognizing aa 19-34 in the N-terminus of Cavβ1. Data are representative of n=3 independent experiments. (**C-E**) Mouse CD4+ T cells were transduced with two *Cacnb1*-specific shRNAs or a control shRNA, and restimulated 4 days later with anti-CD3+CD28 antibodies. **(C)** Cells counts shown are the ratios of sh*Cacnb1* and shCtrl transduced T cells normalized to non-transduced T cells. (**D**) Quantification of the percentage T cells that have undergone cell division (measured by CFSE dilution) at 1 and 3 days after re-stimulation. **(E)** Representative flow cytometry plots (left) and quantification (right) of apoptosis measured by annexin V staining 3 days after re-stimulation. Data in (C-E) are the mean ± SEM of n=7, n=6 and n=8 mice in (C, D, E), respectively. (**F**) Adoptive transfer of CD4+ T cells from SMARTA mice that had been transduced with a *Cacnb1*-specific shRNA or a control shRNA followed by $LCMV^{ARM}$ infection of host mice. Transduced donor T cells were mixed at 1:1 ratio before injection. At day 7 post-infection, the ratios of sh*Cacnb1* / shCtrl T cells (and shCtrl / shCtrl) were analyzed. Representative flow cytometry plots (bottom left) and quantification (bottom right) of T cell ratios. Data are the mean ± SEM from n=3 SMARTA donor mice and n=7 WT host mice per group. Statistical analysis in (A,D, E) was conducted by two-tailed, unpaired Student's *t* test. ***p*<0.01, ****p*<0.001.

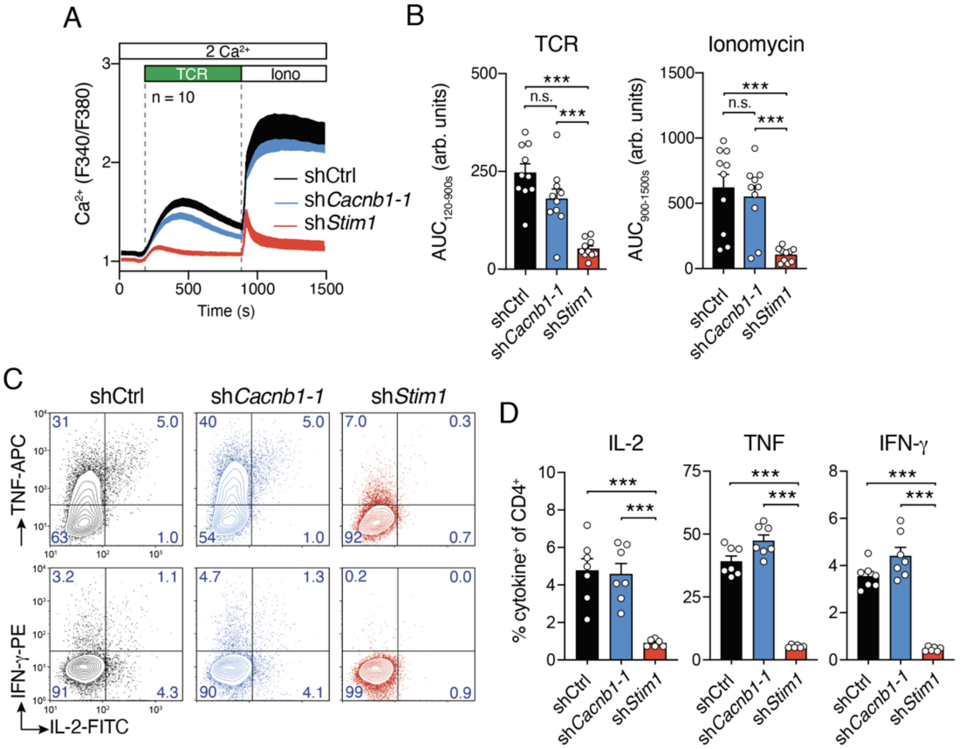

Supplementary Figure 3. Cavβ1 is not required for Ca^{2+} influx and cytokine production by T cells. $CD4^+$ T cells of wildtype mice were transduced with *Cacnb1*-specific, *Stim1*-specific or control shRNAs. (**A**,**B**) After 3 days, Amt^+ T cells were enriched by cell sorting, recovered for one day in medium containing IL-2 and IL-7 and analyzed. Cytosolic Ca^{2+} levels were analyzed following stimulation of T cells by anti-CD3 (TCR) cross-linking and ionomycin (**I**ono) in Ringer's solution containing 2 mM Ca^{2+}. Averaged Ca^{2+} traces (A) and quantification (B) of the area under the curve (AUC) in the time periods indicated by the dotted lines. (**C**,**D**) Cytokine production by $CD4^+$ T cells was measured at day 4 after transduction and restimulation for 6h with PMA and ionomycin. Representative contour plots (C) and quantification (D) of IL-2^+, TNF^+ and IFN-γ^+ $CD4^+$ T cells. Data in (A,B, and D) are the mean ± SEM of n=10 individual experiments. Statistical analysis by two-tailed, unpaired Student's *t* test. ****p*<0.001.

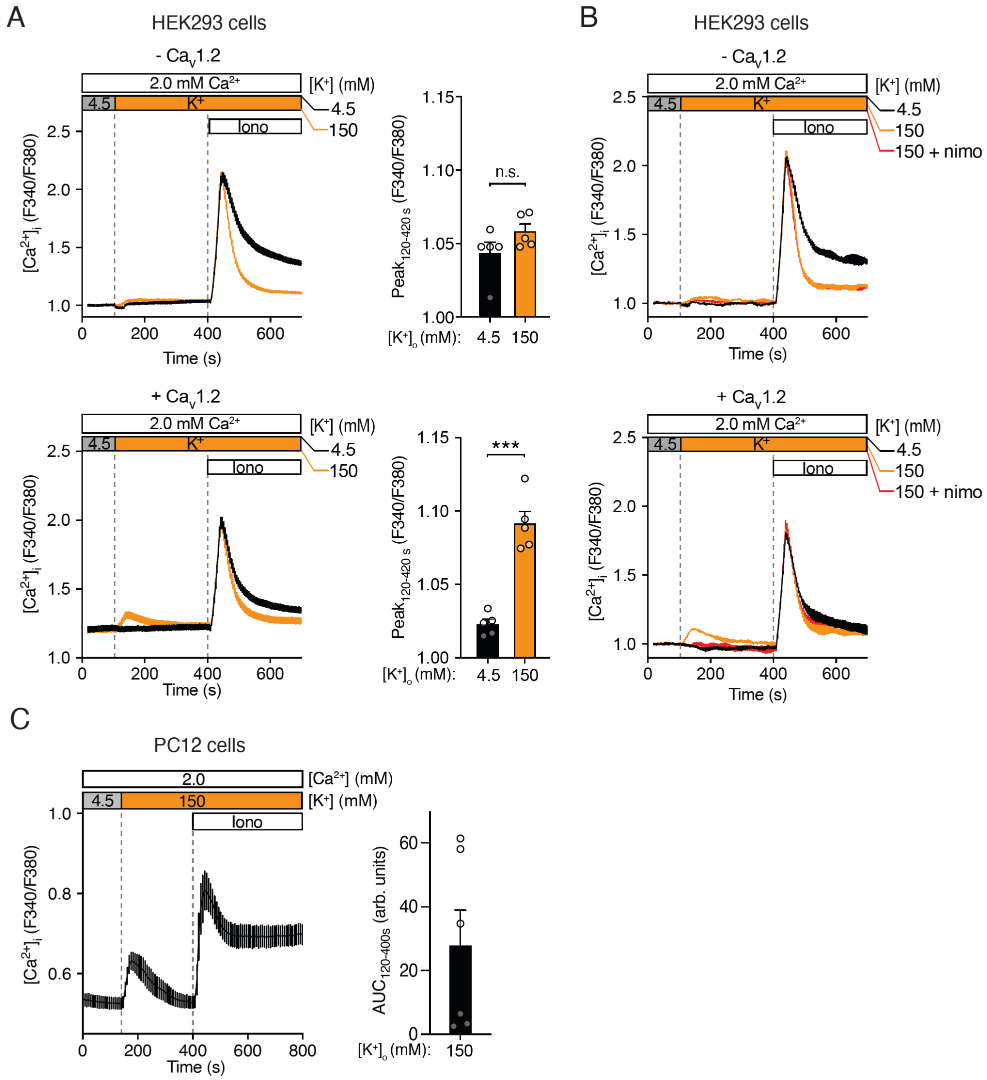

Supplementary Figure 4. Depolarization evokes Ca^{2+} influx in Cav1.2 transfected HEK293 cells and PC12 neuroendocrine cells. (A) Cytosolic Ca^{2+} levels in HEK293 cells untransfected (top) and transfected with α1 subunit of Cav1.2, and β, γ and α2δ subunits (bottom panels). Fura-2 loaded cells were exposed to 150 mM K^+ in the extracellular buffer to induce membrane depolarization. At the end of the experiment, cells were stimulated with 1 μM ionomycin (Iono). Representative Ca^{2+} traces (left) and quantification (right) of the peak F340/F380 ratios after application of 4.5 or 150 mM K^+ buffer. **(B)** Same experimental conditions as in (A), but cells were treated with 8 μM nimodipine to block L-type Ca^{2+} channels for 5 min prior to begin of the recording. Cells were kept in 8 μM nimodipine during the recording. Data in (A) are the mean ± SEM of n=5 independent experiments. Data in (B) are the mean ± SEM from one experiment conducted in quadruplicates. **(C)** Cytosolic Ca^{2+} levels in PC12 cells. Fura-2 loaded cells were treated with 150 mM K^+ in the extracellular buffer followed by the application of 1 μM ionomycin. Representative Ca^{2+} trace (left) and quantification (right) of the area under the curve (AUC) of F340/F380 ratios in the time period indicated by the dotted lines. Data in (C) are the mean ± SEM of n=6 independent experiments. Statistical analysis in (A) by two-tailed, unpaired Student's *t* test. ***p<0.001.

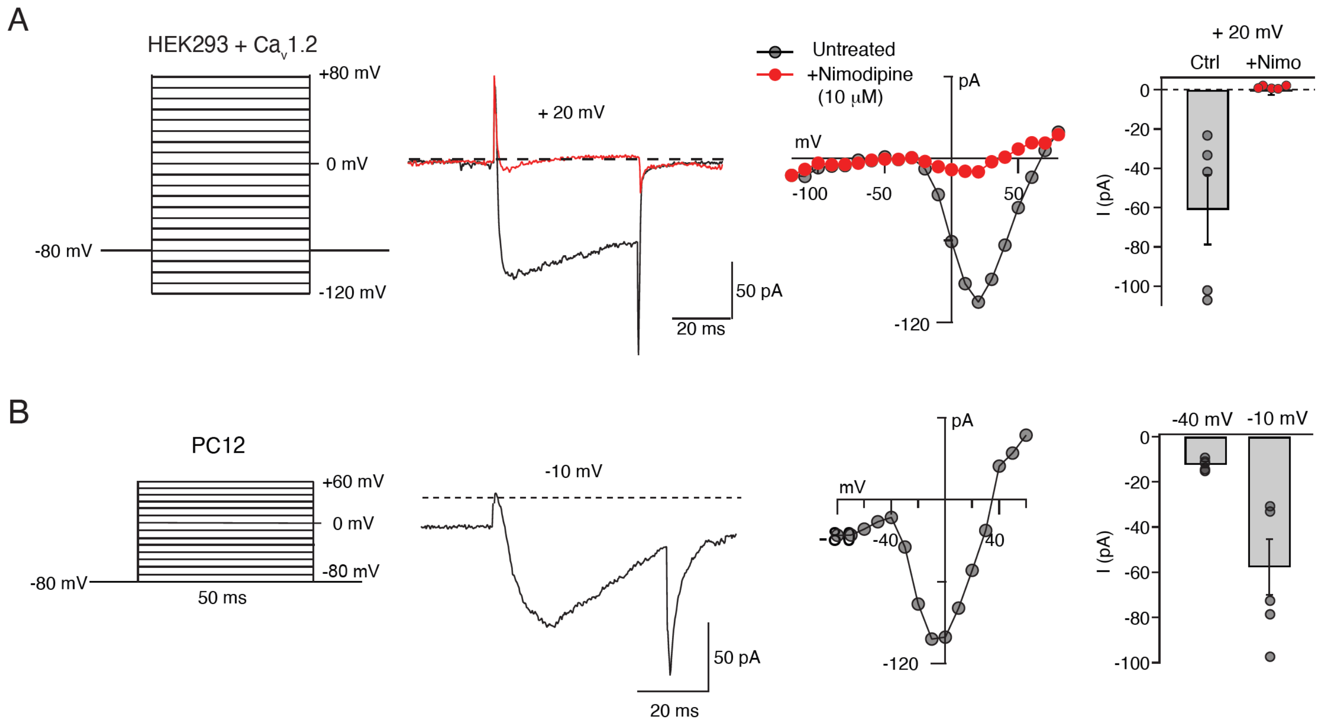

Supplementary Figure 5. Depolarization evokes voltage-gated Ca^{2+} currents in Cav1.2 transfected HEK293 cells and PC12 neuroendocrine cells. (A) HEK293 cells were transfected with Cav1.2. β2, and α2δ1 subunits together with mCherry C1 for visualizing the transfected cells. Representative traces of VGCC currents in a HEK293 cell overexpressing Cav1.2 in the absence (black trace) and presence of 10 μM nimodipine (red trace). The membrane voltage was stepped from -120 to +80 mV in increments of 10 mV from a holding potential of -80 mV. The **I**-V plot on the right shows currents in the presence and absence of nimodipine. The bar graph summarizes the current amplitudes of Cav1.2 in the presence and absence of nimodipine. **(B)** Representative trace of the endogenous VGCC current in PC12 cells. The membrane voltage was stepped from -80 mV (holding) to -10 mV. The **I**-V plot (middle) shows voltage gated Ca^{2+} currents, whose amplitudes at -40 and -10 mV are summarized in the bar graphs on the right. Currents were leak-subtracted by blocking VGCC currents with $LaCl_3$ (100 μM). Data in (A,B) are representative of n=5 cells per condition; bar graphs represent the mean ± SEM.

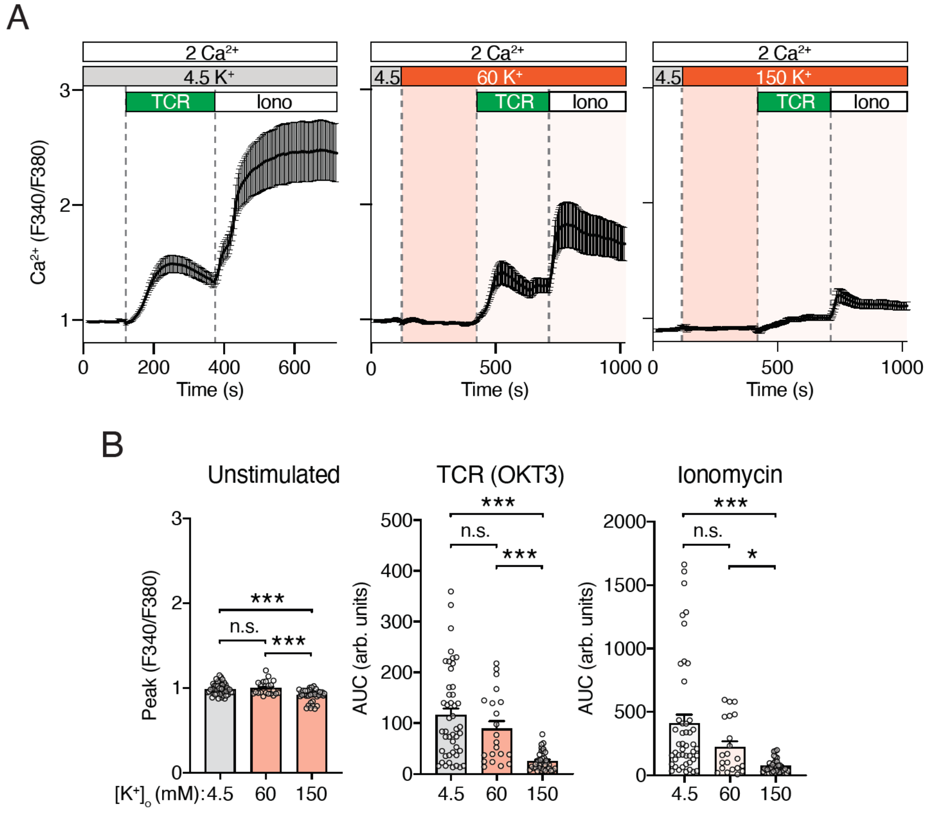

Supplementary Figure 6. Lack of voltage-dependent Ca^{2+} influx in activated human T cells. (A,B) Cytosolic Ca^{2+} levels in $CD4^+$ T cells of human T cells from a healthy donor (HD) cultured for 10 days *in vitro*. Fura-2 loaded T cells were bathed in Ringer's solution containing 60 mM or 150 mM KCl to depolarize cells, followed by TCR crosslinking with anti-CD3 (OKT3) antibodies and stimulation with 1 μM ionomycin (Iono). **(A)** Averaged Ca^{2+} traces and **(B)** quantification of the peak F340/F380 ratio (left graph) and the area under the curve (AUC) of F340/F380 ratios after TCR crosslinking (OKT3) and ionomycin stimulation during the time periods indicated by the dotted lines (middle and left graphs). Data in (A) and (B) are the mean ± SEM of n=11, n=6, and n=5 independent experiments for 4.5, 60 and 150 mM KCl, respectively. Statistical analysis in (B) by two-tailed Mann Whitney U test. *p<0.05, ***p<0.001.

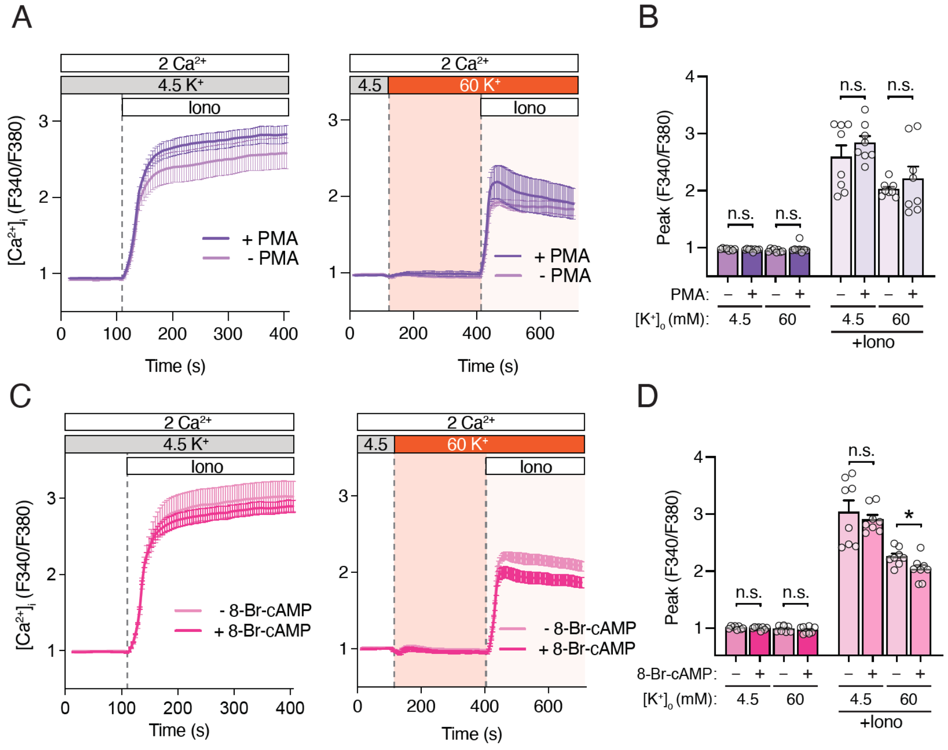

Supplementary Figure 7. Stimulation of PKA or PKC does not evoke voltage-activated Ca^{2+} influx in T cells. (A-D) Cytosolic Ca^{2+} levels were measured in human T cells from a HD cultured for 10 days *in vitro*. T cells were loaded with Fura-2 and left untreated (-) or pretreated (+) for 10 min with 200 nM PMA **(A,B)** or 1 mM 8-Br-cAMP **(C,D)** prior to Ca^{2+} measurements. T cells were kept in Ringer's solution containing 4.5 mM KCl or exposed to Ringer's solution containing 60 mM KCl. Ionomycin stimulation (Iono) was used as positive control. **(A,C)** Averaged Ca^{2+} traces and **(B,D)** quantification of the peak F340/F380 ratios during the indicated recording periods. Data represent the mean ± SEM of n=2 independent experiments conducted in duplicates. Statistical analysis by two-tailed, unpaired Student's *t* test. $^*p<0.05$.

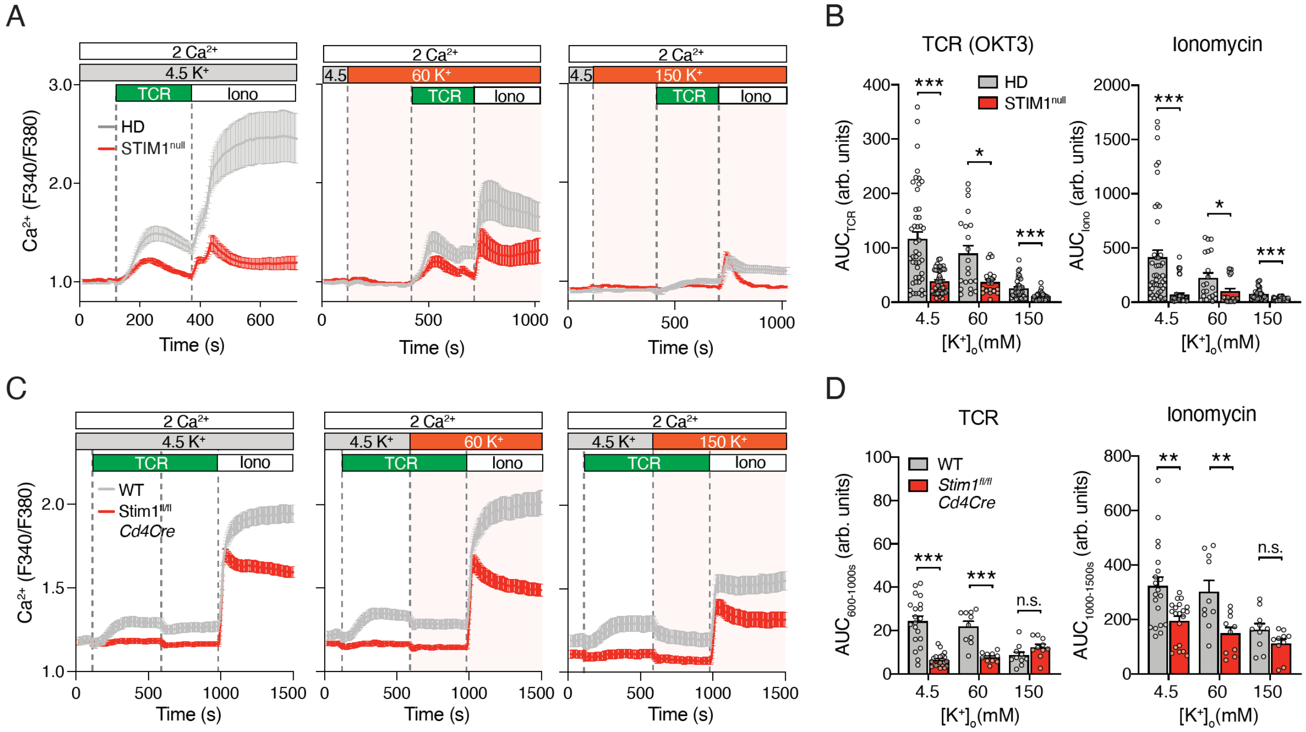

Supplementary Figure 8. Lack of STIM1 does not reveal depolarization-activated Ca^{2+} influx in T cells. (A,B) Cytosolic Ca^{2+} signals in human T cells from a healthy donor (HD, grey) and a patient with a *STIM1* c.497+776A>G null mutation ($STIM1^{null}$) that were cultured for 10 days *in vitro.* Fura-2 loaded T cells were bathed in Ringer's solution containing 60 mM or 150 mM KCl to depolarize cells, followed by TCR crosslinking with anti-CD3 (OKT3) antibodies and stimulation with 1 μM ionomycin (Iono). **(A)** Averaged Ca^{2+} traces and **(B)** quantification of the AUC of F340/F380 ratios during the time periods indicated by the dotted lines. Data are the mean ± SEM of n=11, n=6, and n=5 independent experiments for HD and $STIM1^{null}$ T cells in 4.5, 60 and 150 mM KCl, respectively. (Note that Ca^{2+} traces of HD T cells are the same as those shown in Supplementary Figure 4A; HD T cells were analyzed together with $STIM1^{null}$ T cells and are shown for comparison). **(C,D)** Cytosolic Ca^{2+} signals in $CD4^{+}$ T cells isolated from WT (gray) and *Stim1$^{fl/fl}$ Cd4Cre* mice (red). Fura-2 loaded T cells were stimulated by CD3-crosslinking (TCR) followed by exposure to Ringer's solution containing 60 mM or 150 mM KCl to depolarize cells. Stimulation with 1 μM ionomycin was used as positive control. **(C)** Averaged Ca^{2+} traces and **(D)** quantification of the AUC of F340/F380 ratios during the time periods indicated by the dotted lines. Data are the mean ± SEM from n=4 independent experiments conducted in duplicates. Statistical analysis by two-tailed Mann Whitney U test. *p<0.05, **p<0.01, ***p<0.001.

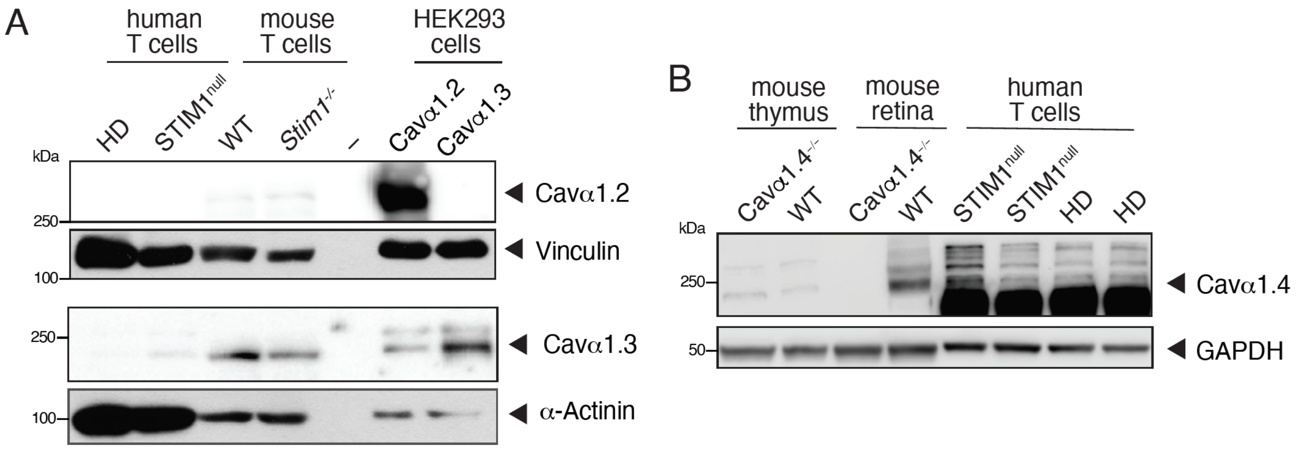

Supplementary Figure 9. Protein expression of α1 subunits of VGCCs in T cells. (A,B) Protein expression of Cav1.2, Cav1.3 **(A)** and Cav1.4 **(B)** in human T cells from a healthy donor (HD), a patient with a *STIM1* c.497+776A>G null mutation (STIM1^{null}) and T cells from WT and *Stim1*$^{fl/fl}$ *Cd4Cre* (*Stim1*$^{-/-}$) mice. Reference samples include thymus and retina from WT and *Cav1.4*$^{-/-}$ mice, and HEK293 cells transfected with Cav1.2 and Cav1.3 mammalian overexpression plasmids. GAPDH, Actin and Vinculin were used as loading controls. Representative Western blots from n=4 independent experiments in A and n=3 independent experiments in (B).

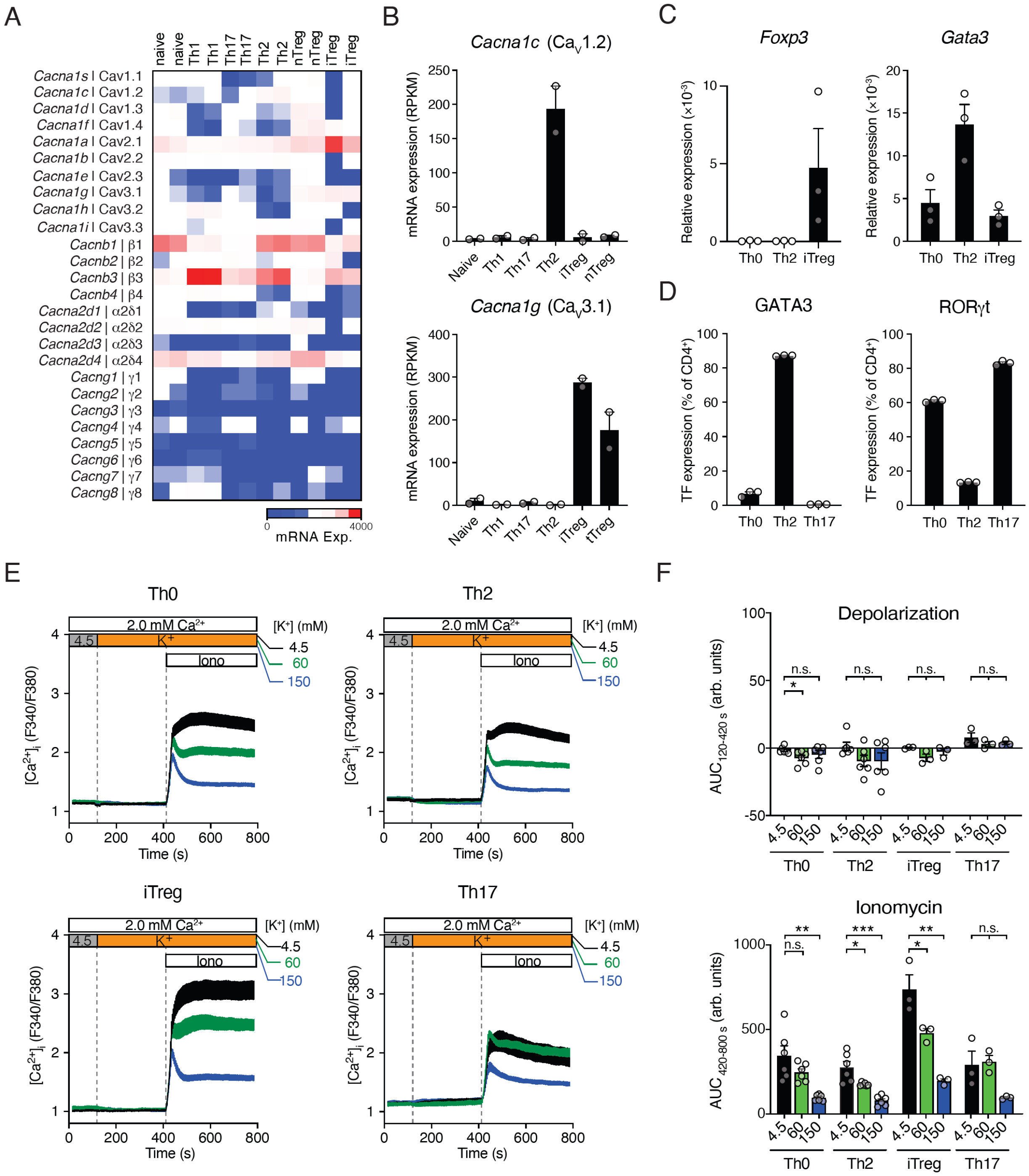

Supplementary Figure 10. Mouse Th2 and Th17 cells lack depolarization-induced Ca^{2+} influx. (A) Heatmap of absolute mRNA expression levels (determined by RNA-Seq) of α_1, β, γ and α2δ subunits of VGCCs in mouse CD4⁺ T cells before (naïve) and after differentiation into Th1, Th2, Th17 and iTreg cells *in vitro* as well as natural Treg (nTreg) cells isolated from the spleen. **(B)** mRNA expression of *Cacna1c* (encoding Cav1.2, top panel) and *Cacna1g* (Cav3.1, bottom panel) in different mouse CD4⁺ T cell subsets. Data in (B) are the mean ± SEM of n=2 biological replicates. Data in (A,B) were reanalyzed from Stubbington et al [1]. **(C-F)** Cytosolic Ca^{2+} levels in mouse CD4⁺ T cells that were differentiated *in vitro* for 3-5 days into Th2 cells (IL-4, anti-IFN-γ, anti-IL-12), Th17

(continued on next page)

(continued from previous page)

cells (anti-IL-4, anti-IFN-γ, IL-6, TGF-β) and iTreg cells (anti-IL-4, anti-IFN-γ, IL-2, TGF-β), and Th0 (anti-IFNγ, anti-IL-4). **(C)** *Foxp3* and *Gata3* mRNA expression in Th0, Th2 and iTreg cells determined by qPCR. *Rlp32* was used as a housekeeping control. **(D)** Frequencies of GATA3 and RORγt expressing Th0, Th2 and iTreg cells determined by flow cytometry. Data in (C,D) are from n=3 independent experiments conducted in triplicates. **(E)** Fura-2 loaded T cells were exposed to 60 mM or 150 mM K^+ in the extracellular buffer to induce membrane depolarization. At the end of the experiment, cells were stimulated with 1 μM ionomycin (Iono). Data are from n=3 independent experiments conducted in triplicates. **(F)** Quantification of the Ca^{2+} responses shown in (E) as the area under the curve (AUC) following application of 60 or 150 mM K^+ (upper panel) and stimulation with ionomycin (lower panel). Data in (F) are the mean ± SEM of at least n=3 independent experiments. Statistical analysis in (F) by two-tailed, unpaired Student's *t* test. *, $p<0.05$; **, $p< 0.01$; ***$p<0.001$.

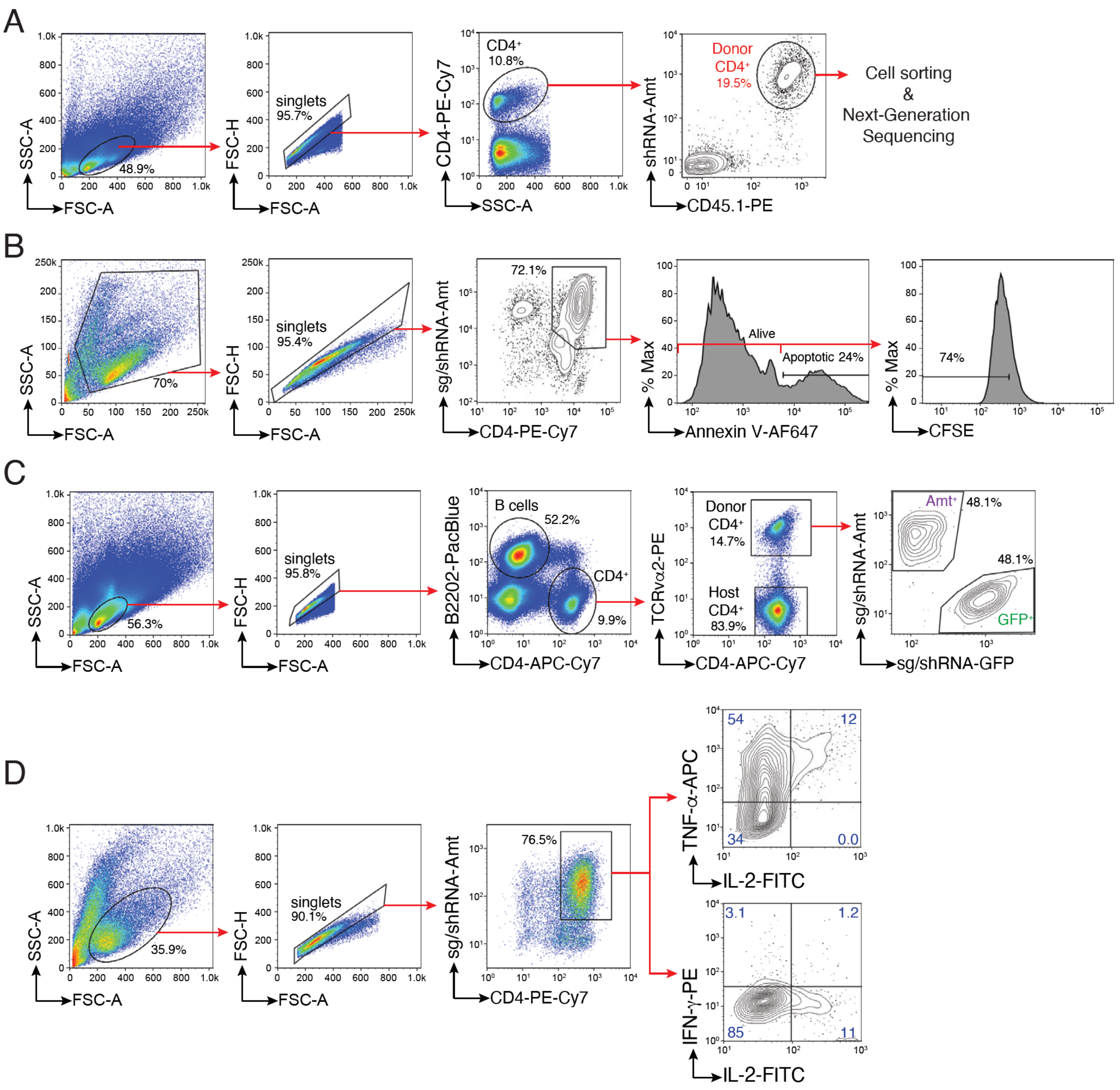

Supplementary Figure 11. Flow cytometry analyses and gating strategies. (A-D) Representative plots showing the gating strategy used for flow cytometry analysis in Figure 1A (in A), Figures 2D,E and Supplementary Figures 2D,E (in B), Figure 2F and Supplementary Figure 2F (in C), and Figure 3C and Supplementary Figure 3C (in D).

SUPPLEMENTARY TABLES

Supplementary Table 1. Recombinant DNA

Reagent or Resource	Retroviral vector	Target sequence (for sh or sgRNA)
shCacnb1 (mouse)	pLMPD-Ametrine	sh1: TTATGTCAAACATTTCGGGGGG<br>sh2: TTGATGTAAACAATGATGGGGG
shStim1 (mouse)	pLMPD-Ametrine	ATGCGGTTTCCAGATTGTCAAT
shRenilla.713	pLMPD-Ametrine or pLMPD-GFP	TAGATAAGCATTATAATTCCTA
sgCacnb1 (mouse)	MRI-Ametrine	GTCTGATGGTCGGCTCGTGTGT
sgStim1 (mouse)	MRI-Ametrine	TGAGGATAAGCTTATCAGCG
sgVEGF (human)	MRI-Ametrine or MRI-GFP	GAGCAGCGTCTTCGAGAGTG

Supplementary Table 2. RNA-Seq datasets

Source	Species	Cell / Tissue	Figure	Ref.
GSE49366	*M.m.*	$CD4^+$ T cells	Figs 1E, 7B, 7G, 7H	PMID: 25368162
GSE106463	*M.m.*	$CD4^+$ T cells	Figs 1E, 7B, 7G, 7H	PMID: 29662170
GSE96724	*M.m.*	$CD4^+$ T cells	Figs 1E, 7B, 7G, 7H	PMID: 28623086
GSE52260	*M.m.*	$CD4^+$ T cells	Figs 1E, 7B, 7G, 7H	PMID: 26967054
GSE79219	*M.m.*	$CD8^+$ T cells	Figs 1E, 7B	
GSE84927	*M.m.*	Retina	Figs 1E, 7B	PMID: 28172828
GSE120423	*M.m.*	Brain	Figs 1E, 7B, 7G, 7H	PMID: 31053723
GSE99522	*M.m.*	Heart	Figs 1E, 7B	PMID: 30102730
GSE130838	*M.m.*	Skeletal Muscle	Figs 1E, 7B	PMID: 31110021
E-MTAB-2582	*M.m.*	$CD4^+$ T cells	Supplementary Figure 10A,B	PMID: 25886751
GSE179625	*H.s.*	$CD4^+$ T cells	Figures 1F, 7A, 7C, 7D, 7E, 7F	This publication
GSE46224	*H.s.*	Heart	Figs 1F, 7A	PMID: 24429688
GSE58387	*H.s.*	Skeletal Muscle	Figs 1F, 7A	PMID: 25016029
GSE115828	*H.s.*	Retina	Figs 1F, 7A	PMID: 30742112
GSE64810	*H.s.*	Frontal Cortex	Figs 1F, 7A, 7C, 7D, 7E, 7F	PMID: 26636579
GSE87508	*H.s.*	T cells	Suppl Figs 1E, 1F	PMID: 31308016
GSE133822	*H.s.*	T cells	Suppl Figs 1E, 1F	PMID: 31484735

Supplementary Table 3. Antibodies used for flow cytometry

Antigen	Source	Clone	Conjugation	Intracellular staining
Human CD4	BioLegend	RPA-T4	PE, PE/Cy7	n/a
Human CD8	BioLegend	SK1	FITC	n/a
Mouse CD4	BioLegend	GK1.5	PE/Cy7, APC/Cy7	n/a
Mouse CD45.1	eBioscience	A20	PE	n/a
Mouse TCRvα 2	eBioscience	B20.1	PE	n/a
Mouse IL-2	Invitrogen	JES6-5H4	FITC	IC staining Kit (eBioscience)
Mouse IFN-γ	BioLegend	XMG1.2	PE	IC staining Kit (eBioscience)
Mouse TNF-α	eBioscience	MP6-XT22	APC	IC staining Kit (eBioscience)
Mouse RORγt	eBioscience	B2D	PE	TF staining kit (eBioscience)
Human/Mouse Gata3	eBioscience	TWAJ	eFluor660	TF staining kit (eBioscience)

Supplementary Table 4. Primers used for qRT-PCR

Gene name	Forward primer	Reverse primer
Mouse *Cacnb1*	TGTAGCCAACATTTGTCCGA	CGAAGCCTTAAGGAAGGAGG
Mouse *FoxP3*	CACCCAGGAAAGACAGCAACC	GCAAGAGCTCTTGTCCATTGA
Mouse *Gapdh*	AAGCTCATTTCCTGGTATGACA	CTTGCTCAGTGTCCTTGCTG
Mouse *Gata3*	AGGATGTCCCTGCTCTCCTT	GCCTGCGGACTCTACCATAA
Mouse *Stim1*	ATTCGGCAAAACTCTGCTTC	GGCCAGAGTCTCAGCCATAG
Mouse *Rpl32*	CCCAACATCGGTTATGGGAGCA	GATGGCCAGCTGTGCTGC

SUPPLEMENTARY REFERENCES

1 Stubbington, M. J. *et al.* An atlas of mouse CD4(+) T cell transcriptomes. *Biol Direct* **10**, 14, doi:10.1186/s13062-015-0045-x (2015).
