# Supplementary material for: Cavβ1 regulates T cell expansion and apoptosis independently of voltage-gated Ca2+ channel function
Source: Nat Commun. 2022 Apr 19;13:2033. doi: 10.1038/s41467-022-29725-3 (PMC9018955; doi:10.1038/s41467-022-29725-3)
Supplement: Supplementary file 3 — Reporting Summary [file 41467_2022_29725_MOESM3_ESM.pdf]

nature research

Corresponding author(s): Stefan Feske
Last updated by author(s): 2022/02/07

Reporting Summary

Nature Research wishes to improve the reproducibility of the work that we publish. This form provides structure for consistency and transparency in reporting. For further information on Nature Research policies, see our Editorial Policies and the Editorial Policy Checklist.

Statistics

For all statistical analyses, confirm that the following items are present in the figure legend, table legend, main text, or Methods section.

n/a	Confirmed	
☐	☒	The exact sample size (*n*) for each experimental group/condition, given as a discrete number and unit of measurement
☐	☒	A statement on whether measurements were taken from distinct samples or whether the same sample was measured repeatedly
☐	☒	The statistical test(s) used AND whether they are one- or two-sided<br>*Only common tests should be described solely by name; describe more complex techniques in the Methods section.*
☒	☐	A description of all covariates tested
☐	☒	A description of any assumptions or corrections, such as tests of normality and adjustment for multiple comparisons
☐	☒	A full description of the statistical parameters including central tendency (e.g. means) or other basic estimates (e.g. regression coefficient) AND variation (e.g. standard deviation) or associated estimates of uncertainty (e.g. confidence intervals)
☐	☒	For null hypothesis testing, the test statistic (e.g. *F*, *t*, *r*) with confidence intervals, effect sizes, degrees of freedom and *P* value noted<br>*Give P values as exact values whenever suitable.*
☒	☐	For Bayesian analysis, information on the choice of priors and Markov chain Monte Carlo settings
☒	☐	For hierarchical and complex designs, identification of the appropriate level for tests and full reporting of outcomes
☐	☒	Estimates of effect sizes (e.g. Cohen's *d*, Pearson's *r*), indicating how they were calculated

Our web collection on statistics for biologists contains articles on many of the points above.

Software and code

Policy information about availability of computer code

Data collection

Electrophysiology data was collected by using Axopatch 200 amplifier (Axon Instruments) interfaced to an ITC-16 input/output board (Instrutech) and a Macintosh G3 computer. Data acquisition and analysis were performed using in-house routines (R. Lewis, Stanford University) developed on the Igor Pro platform (Wavemetrics).

Time-lapse imaging data was acquired using SlideBook imaging software (v4.2) on an Olympus IX81 inverted epifluorescence microscope (Olympus) equipped to Lambda DG-4 Plus light source and wavelength switcher (Sutter Instruments). Some time-lapse imaging experiments were performed simultaneously with patch-clamping electrophysiology using a Nikon Diaphot TMD microscope equipped with a Nikon Fluor objective (NA 1.3).

Calcium measurements were acquired using SoftMax Pro v5.4.6 software in the FlexStation 3 Multi-Mode Microplate Reader (Molecular Devices).

Flow cytometry data was acquired using FACSDiva in the BD LSR Fortessa Flow Cytometer (BD Bioscience).

RNA-Seq libraries were quantified by Qubit 2.0 fluorometer (Life Technologies), and visualized using an Agilent Tapestation 2200. The libraries were pooled equimolarly, loaded on the HiSeq 2500 DNA Sequencer and run as single 50 nucleotide reads

Immunoblot data was collected using Odyssey Fc Western Blot Detection System (Licor Bioscience) for immunofluorescent bands and an Amersham Imager 680 (GE) for protein detection using chemiluminescence.

Quantitative Real Time PCR data was acquired using a QuantStudio 3 PCR machine (Applied Biosystem, Thermo Fisher Scientific).

Data analysis

Sequencing results from pooled shRNA screening were analyzed using the Model-based Analysis of Genome-wide CRISPR-Cas9 Knockout (MAGeCK) package (PMID: 25476604) to identify shRNAs that were depleted or enriched in donor T cells after LCMV infection compared to input T cell samples.

For gene expression analysis, the FASTQ files from RNA-Seq datasets generated in this study or extracted from the Gene Expression Omnibus (GEO) database were trimmed with Trimmomatic (v0.36) (PMID: 24695404) and aligned with STAR (v2.6.1) (PMID: 23104886) to the corresponding human GRCh38/hg38 and mouse GRCh38/mm10 genome assembly. Alignments were guided by a Gene Transfer Format file (GTF). The read count tables for exon level data were generated using HTSeq (v0.11.2) (PMID: 25260700), normalized based on their library size factors using DEseq2 (PMID: 25516281). The Reads Per Kilobase of transcript per Million reads (RPKM) normalized BigWig files were generated using deeptools (v3.1.0) (PMID: 27079975). The visualization of BigWig files are generated from aligned reads after performing RPKM normalization was done using pyGenomeTracks (PMID: 32745185). Human gene level transcripts were counted using Salmon (v0.14.1) (PMID: 28263959) with annotation from Gencode (v30) Gene Transfer Format (GTF) file, and mouse gene level transcripts were aligned and counted using the featureCounts function in the subread package (v1.6.3) (PMID: 24227677) with annotation from Gencode Gene Transfer M21 (GTF GRCm38.p6) format file . The final data to generate heatmaps of gene expression for human and mouse samples was TPM normalized to allow comparison of expression levels between different genes. The final heatmap visualization was done in python using the mwaskom/seaborn:v0.8.1 package (M. Waskom et al. (2017) mwaskom/seaborn: v0.8.1 Zenodo).

For exon-usage analysis, mRNA gene expression from human and mouse tissues and cells was performed using the HTSeq package (v0.11.2) (PMID: 25260700) with annotations from the human (GRCh38.p12) Gencode v30 GTF file and mouse (GRCm38.p6) Gencode M25 GTF file GTF, respectively. The exon-level RNA expression data was normalized using the median of ratios method from DESeq2 (PMID: 25516281) Bioconductor package in R (version 3.6.1). Bars graphs showing quantification of DESeq2 normalized mRNA expression for each exon were generated using MATLAB R2019. The visualization of bigWig files generated from aligned reads after performing RPKM normalization was done using pyGenomeTracks (PMID: 32745185). For transcriptional start site (TSS) analysis, Potential TSS near the 5' end of mRNA transcripts in T cells were obtained from refTSS (PMID: 31075273).

Flow cytometry data was analyzed using the FlowJo 10.5.3 software (BD Bioscience).

Quantitative Real Time PCR data was analyzed using a QuantStudio Design & Software Analysis (Applied Biosystem, Thermo Fisher Scientific).

Band densities from Immunoblots were quantified and analyzed using ImageJ 1.52a.

All data was visualized with Microsoft Excel v 16.51 and Graphpad Prism and statistical analysis was done using the Prism Software (Graphpad) v 9.0.2.

For manuscripts utilizing custom algorithms or software that are central to the research but not yet described in published literature, software must be made available to editors and reviewers. We strongly encourage code deposition in a community repository (e.g. GitHub). See the Nature Research guidelines for submitting code & software for further information.

Data

Policy information about availability of data

All manuscripts must include a data availability statement. This statement should provide the following information, where applicable:

- Accession codes, unique identifiers, or web links for publicly available datasets
- A list of figures that have associated raw data
- A description of any restrictions on data availability

The RNA-Seq data generated in this study have been deposited in the GEO database and SRA data repositories with GSE179625 (https://www.ncbi.nlm.nih.gov/geo/query/acc.cgi?acc=GSE179625). The RNA-Seq data from human and mouse tissues extracted from the GEO database are listed in Supplemental Table 2. Additionally, gene expression datasets for the Immunological Genome Project (GSE15907, PMID: 18800157, https://www.ncbi.nlm.nih.gov/geo/query/acc.cgi?acc=GSE15907) and Haemopedia (GSE115736 & GSE116177, PMID: 30395284, www.haemosphere.org) were downloaded from the Sequence Read Archive (SRA). Datasets for Fantom5 (https://fantom.gsc.riken.jp/5/, human CAGE-Seq (GSE49834, PMID: 24670764).

Additional RNA-Seq datasets from published sources have been listed in Supplemental Table 2 and referenced in Data Availability section:
Accession codes / Species, Cell / Tissue / Figure / Reference (PMID)/ Link
GSE49366 , https://www.ncbi.nlm.nih.gov/geo/query/acc.cgi?acc=GSE49366
GSE106463, https://www.ncbi.nlm.nih.gov/geo/query/acc.cgi?acc=GSE106463
GSE96724, https://www.ncbi.nlm.nih.gov/geo/query/acc.cgi?acc=GSE96724
GSE52260, https://www.ncbi.nlm.nih.gov/geo/query/acc.cgi?acc=GSE52260
GSE79219, https://www.ncbi.nlm.nih.gov/geo/query/acc.cgi?acc=GSE79219
GSE84927, https://www.ncbi.nlm.nih.gov/geo/query/acc.cgi?acc=GSE84927
GSE120423, https://www.ncbi.nlm.nih.gov/geo/query/acc.cgi?acc=GSE120423
GSE99522, https://www.ncbi.nlm.nih.gov/geo/query/acc.cgi?acc=GSE99522
GSE130838, https://www.ncbi.nlm.nih.gov/geo/query/acc.cgi?acc=GSE130838
E-MTAB-2582, https://www.ebi.ac.uk/arrayexpress/experiments/E-MTAB-2582/
GSE179625, https://www.ncbi.nlm.nih.gov/geo/query/acc.cgi?acc=GSE179625
GSE46224, https://www.ncbi.nlm.nih.gov/geo/query/acc.cgi?acc=GSE46224
GSE58387, https://www.ncbi.nlm.nih.gov/geo/query/acc.cgi?acc=GSE58387
GSE115828, https://www.ncbi.nlm.nih.gov/geo/query/acc.cgi?acc=GSE115828
GSE64810, https://www.ncbi.nlm.nih.gov/geo/query/acc.cgi?acc=GSE64810
GSE87508, https://www.ncbi.nlm.nih.gov/geo/query/acc.cgi?acc=GSE87508
GSE133822, https://www.ncbi.nlm.nih.gov/geo/query/acc.cgi?acc=GSE133822

Field-specific reporting

Please select the one below that is the best fit for your research. If you are not sure, read the appropriate sections before making your selection.

[x] Life sciences [ ] Behavioural & social sciences [ ] Ecological, evolutionary & environmental sciences

For a reference copy of the document with all sections, see nature.com/documents/nr-reporting-summary-flat.pdf

Life sciences study design

All studies must disclose on these points even when the disclosure is negative.

Sample size	No statistical methods were used to calculate sample sizes, but our sample sizes are similar to those reported in previous publications (PMID: 18327260, PMID: 30773462). Furthermore, fundamental findings were confirmed with a variety of different methods to avoid any method-specific bias. Sample sizes were based on experience, experimental complexity. Furthermore, fundamental findings were confirmed with a variety of different methods to avoid any method-specific bias. In addition, each figure legend contains the information how many samples/cells/mice were used for the described experiment.
Data exclusions	No data were excluded.
Replication	All experiments were done at least in two independent experiments but generally more than 2. The total number of mice and independent experiments performed is indicated in the figure legends. For human T cell experiments, cells from one single patient were assessed at different time points to collect data from independent experiments.
Randomization	Unless when restricted by the genotype, animals and cell plates were assigned randomly to experimental conditions.
Blinding	The Investigators were not blinded to the experimental design as the genotype for human and mouse T cells are already known.

Reporting for specific materials, systems and methods

We require information from authors about some types of materials, experimental systems and methods used in many studies. Here, indicate whether each material, system or method listed is relevant to your study. If you are not sure if a list item applies to your research, read the appropriate section before selecting a response.

Materials & experimental systems

n/a	Involved in the study	
[ ]	[x]	Antibodies
[ ]	[x]	Eukaryotic cell lines
[x]	[ ]	Palaeontology and archaeology
[ ]	[x]	Animals and other organisms
[ ]	[x]	Human research participants
[x]	[ ]	Clinical data
[x]	[ ]	Dual use research of concern

Methods

n/a	Involved in the study	
[x]	[ ]	ChIP-seq
[ ]	[x]	Flow cytometry
[x]	[ ]	MRI-based neuroimaging

Antibodies

Antibodies used

For flow cytometry analysis, the following list of antibodies were used:
Antigen, Clone, Conjugation, Source, all antibodies were used as a 1:200 dilution:
Human CD4, RPA-T4 PE, PE/Cy7, BioLegend
Human CD8, SK1, FITC, BioLegend
Mouse CD4, GK1.5, PE/Cy7, BioLegend
Mouse CD4, GK1.5, APC/Cy7, BioLegend
Mouse CD45.1, A20, PE, eBioscience
Mouse IL-2, JES6-5H4, FITC, Invitrogen
Mouse IFN-g, XMG1.2, PE, BioLegend
Mouse TNF-a, MP6-XT22, APC, eBioscience
Mouse TCRva2, B20.1, PE, eBioscience
Mouse RORgt, B2D, PE, eBioscience
Human/Mouse Gata3, eFluor® 660, TWAJ, eBioscience

For immunoblotting experiments, the following list of antibodies were used:
Cav1.2, rabbit polyclonal, FP1, 1:2000, provided by Johannes W. Hell (PMID: 28781760).
Cav1.3, rabbit polyclonal, 1:500 to 1:1000, provided by Amy Lee (PMID: 20392935).

Cav1.4, rabbit polyclonal, 1:500 to 1:1000, provided by Amy Lee (PMID: 24064553).
Cavβ1, mouse monoclonal antibody, clone N7/18, 1:600, Abcam, ab85020
Mouse anti-β-Actin monoclonal antibody, clone 7D2C10, 1:5000, Proteintech, 66009-1-Ig
Mouse anti-GAPDH, clone 14C10, 1:4000, Cell Signaling Technology, mAb #2118
Vinculin, rabbit plyclonal, 1:4000, Cell Signaling Technology, 4650
HRP-conjugated secondary polyclonal antibody, anti-mouse IgG (Fab specific), 1:5,000 to 1:10,000, Sigma, A9044
IRDye 680RD donkey anti-mouse IgG secondary antibody, 1:10000, LI-COR, 925-68072

For T cell culture experiments, the following list of antibodies were used:
anti-CD3, Bio X cell, clone 2C11, 14-0031-85, 1 ug/ml concentration
anti-CD28, Bio X cell, clone 37.5, BE0015-1, 1 ug/ml concentration
anti-IFN-gamma, eBioscience, clone XMG1.2, 5 ug/ml concentration
anti-IL-4, eBioscience, clone 11B11, 14-7041-81, 5 ug/ml concentration
anti-IL-12, Bio X cell, clone R1-5D9, BE0052, 5 ug/ml concentration

Validation

All antibodies listed above were either purchased commercially or provided by collaborators, validated by manufacturers requirements for process validation documented on their website. Biolegend (https://www.biolegend.com/Files/Images/BioLegend/literature/images/07-0139-02_IHC.pdf), eBioscience, invitrogen (https://www.thermofisher.com/us/en/home/life-science/antibodies/invitrogen-antibody-validation.html), abcam (https://www.abcam.com/primary-antibodies/a-guide-to-antibody-validation), proteintech (https://www.ptglab.com/news/company-news/gen-validating-antibodies-for-specificity/), Cell Signaling (https://www.cellsignal.com/about-us/cst-antibody-validation-principles), Sigma (https://www.sigmaaldrich.com/US/en/technical-documents/technical-article/protein-biology/immunohistochemistry/antibody-enhanced-validation), Bio X cell (https://bxcell.com/performance-guarantee/), LI-COR (https://www.licor.com/bio/guide/westerns/validate_antibodies). All antibodies were tested for their performance in the lab as well.

Eukaryotic cell lines

Policy information about cell lines

Cell line source(s)

Platinum-E retroviral packaging cell line, Cell Biolabs Inc, Catalog Number: RV-101 (https://www.cellbiolabs.com/platinum-e-plat-e-retroviral-packaging-cell-line), Human embryonic kidney 293 (HEK293) cells, ATCC, Catalog Number: CRL-1573 (https://www.atcc.org/products/crl-1573), and PC12 cells, ATCC, Catalog Number: CRL-1721 (https://www.atcc.org/products/crl-1721).

Authentication

Authentication of Plat-E cells was evaluated by resistance to puromycin and blasticidin and by their capacity to produce retroviruses upon lipo-transfection. Cell Morphology, proliferation rate and cell adhesion were evaluated by phase-contrast microscopy. No additional procedures/techniques were used to authenticate additional cell lines.

Mycoplasma contamination

All cells were mycoplasma free tested by PCR.

Commonly misidentified lines
(See ICLAC register)

No commonly misidentified cell lines were used in this study.

Animals and other organisms

Policy information about studies involving animals; ARRIVE guidelines recommended for reporting animal research

Laboratory animals

Stim1fl/fl Cd4Cre (PMID: 18327260), Cacna1f-mutant (PMID: 16155113), congenic CD45.1+ SMARTA (PMID: 29030115) have been described previously. Congenic CD45.2+ (strain 000664), Cd4Cre (strain 017336) and Rosa26-LSL-Cas9 knock-in (strain 024857) mice were purchased from the Jackson laboratory (Bar Harbor, ME). SMARTA; LSL-Cas9; Cd4Cre mice were generated by crossing SMARTA, Cd4Cre and LSL-Cas9 mice. All animals were on a pure C57BL/6 genetic background and housed under Specific Pathogen Free (SPF) conditions with a 12 hour dark and light cycle, 22–25°C, and 50-60% humidity with water and food provided ad libitum. Male and Female mice were used between 8 and 16 weeks of age. All mouse experiments were carried out with sex and age matched groups.

Wild animals

No wild animals were used.

Field-collected samples

No field collected samples were used.

Ethics oversight

Institutional Animal Care and Use Committee (IACUC) of New York University Grossman School of Medicine

Note that full information on the approval of the study protocol must also be provided in the manuscript.

Human research participants

Policy information about studies involving human research participants

Population characteristics	Samples (peripheral blood mononuclear cells) from Healthy donors (HD) and patients with Calcium-Release-Activated Ca2+ channel-(CRAC) channelopathy (PMID: 26469693) were used in this study. The patients with CRAC channelopathy include a 6 month-old male homozygous for an autosomal recessive p.R91W loss-of-function (LOF) mutation in the ORAI1 gene (PMID: 16582901) and a 7 year-old male homozygous for a cryptic splice acceptor site mutation in an intronic region between exons 4 and 5 of STIM1 (c.497+776A>G) which leads to a null mutation in STIM1 (this report and a forthcoming detailed case study). Both mutations abolish store-operated Ca2+ influx (SOCE) in T cells and cause CRAC channelopathy syndrome.
Recruitment	Deidentified patient samples were referred to us by clinicians treating these patients with manifestations of CRAC channelopathy syndrome with the goal to identify the underlying ORAI1 and STIM1 mutations in these patients.
Ethics oversight	Informed consent for the studies was obtained from the patient and healthy donors in accordance with the Declaration of Helsinki and Institutional Review Board approval of the New York University Grossman School of Medicine.

Note that full information on the approval of the study protocol must also be provided in the manuscript.

Flow Cytometry

Plots

Confirm that:

- [x] The axis labels state the marker and fluorochrome used (e.g. CD4-FITC).
- [x] The axis scales are clearly visible. Include numbers along axes only for bottom left plot of group (a 'group' is an analysis of identical markers).
- [x] All plots are contour plots with outliers or pseudocolor plots.
- [x] A numerical value for number of cells or percentage (with statistics) is provided.

Methodology

Sample preparation	Human T cells were separated from whole blood by density gradient centrifugation using Ficoll-Paque plus (GE Amersham) and expanded in vitro as previously described (PMID: 32609955).<br><br>Murine CD4+ T cells were purified from splenocytes using the MagniSort Mouse CD4+ T cell Enrichment Kit (Invitrogen, MS22-7762-74) according to manufacturer's protocol. For pooled shRNA screening and further validation experiments, donor T cells were isolated from the spleen of host mice and purified by cell sorting 7 days after LCMV infection. Staining of cell surface or intracellular antigens with fluorescently-labeled antibodies was carried at room temperature.
Instrument	Samples were acquired on a BD LSR Fortessa Cell Analyzer (BD Biosciences), Model: N/A, SN: R64717700200
Software	Data was analyzed using the FlowJo 10.5.3 software (BD Bioscience).
Cell population abundance	Cell populations were analyzed by gating on live lymphocytes and single cell fractions. Depending on the experimental question relative or absolute numbers were calculated. A representative example of flow cytometry data (dot plots) and the summary of several independent experiments (e.g. bar graphs) is provided in the figures.
Gating strategy	Cell populations were analyzed by gating on live lymphocytes (FSC/SSC) and single cell fractions (FSC-A/FSC-H/FSC-W). Depending on the experimental question, different gating strategies were used and displayed in the respective figure.

- [x] Tick this box to confirm that a figure exemplifying the gating strategy is provided in the Supplementary Information.
