# Supplementary figures and images for: Cavβ1 regulates T cell expansion and apoptosis independently of voltage-gated Ca2+ channel function
Source: Nat Commun. 2022 Apr 19;13:2033. doi: 10.1038/s41467-022-29725-3 (PMC9018955; doi:10.1038/s41467-022-29725-3)

Figure 2b

Original blots

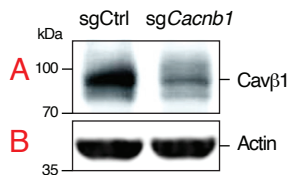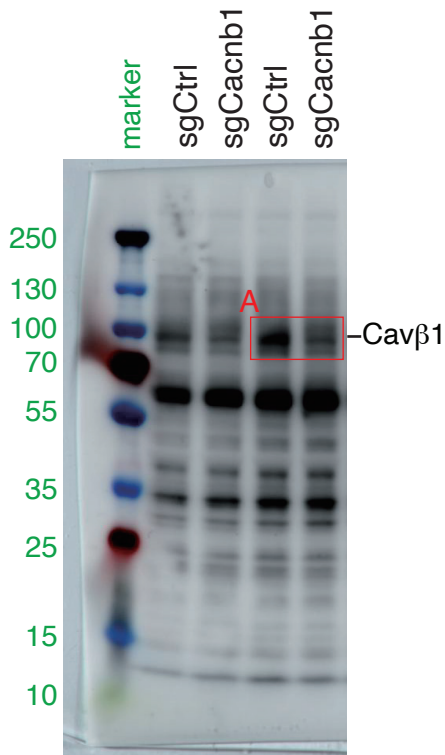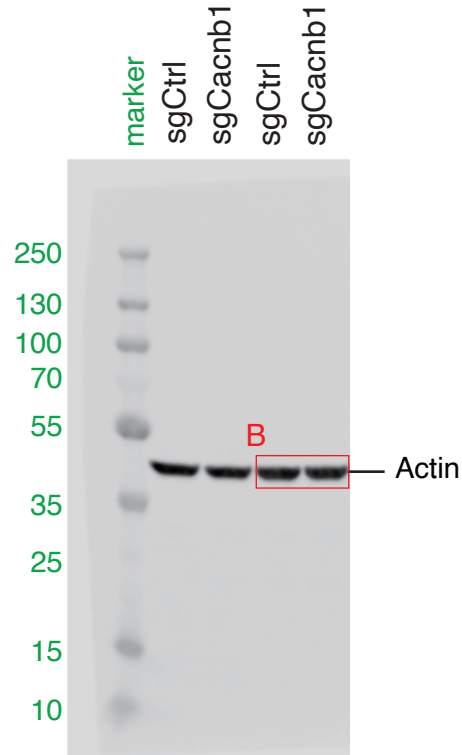

Supplement: Supplementary file 4 — Source Data [file 41467_2022_29725_MOESM4_ESM.zip › Source Data/Figure 2 blots.pdf]

# Supplementary Figure 2b

# Original blots

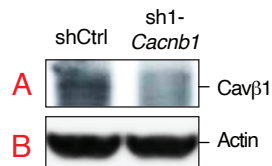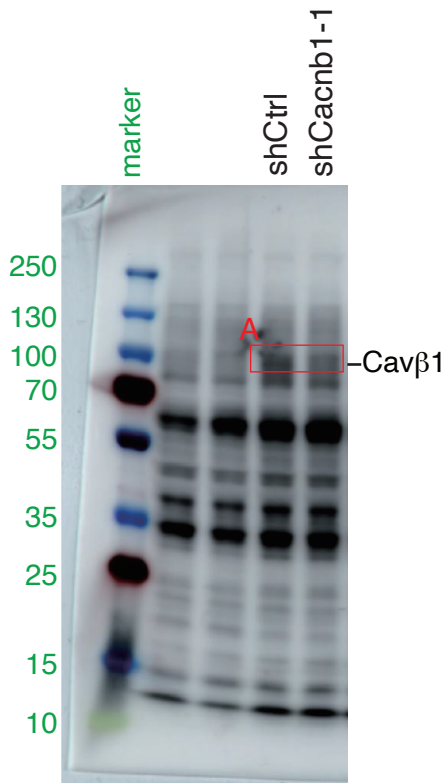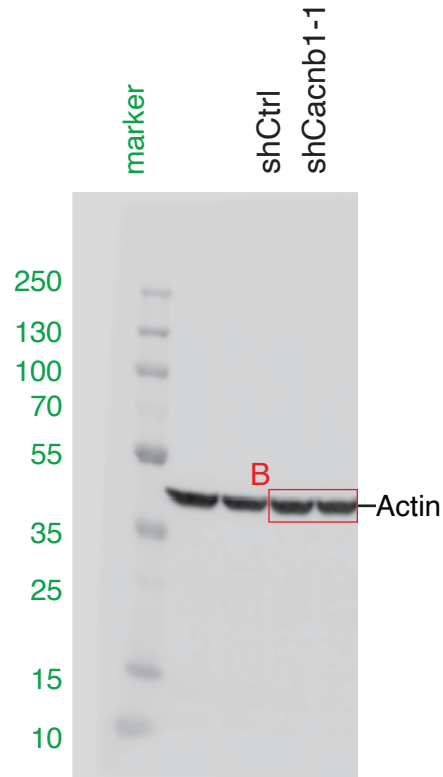

Supplementary Figure 9

Original blots

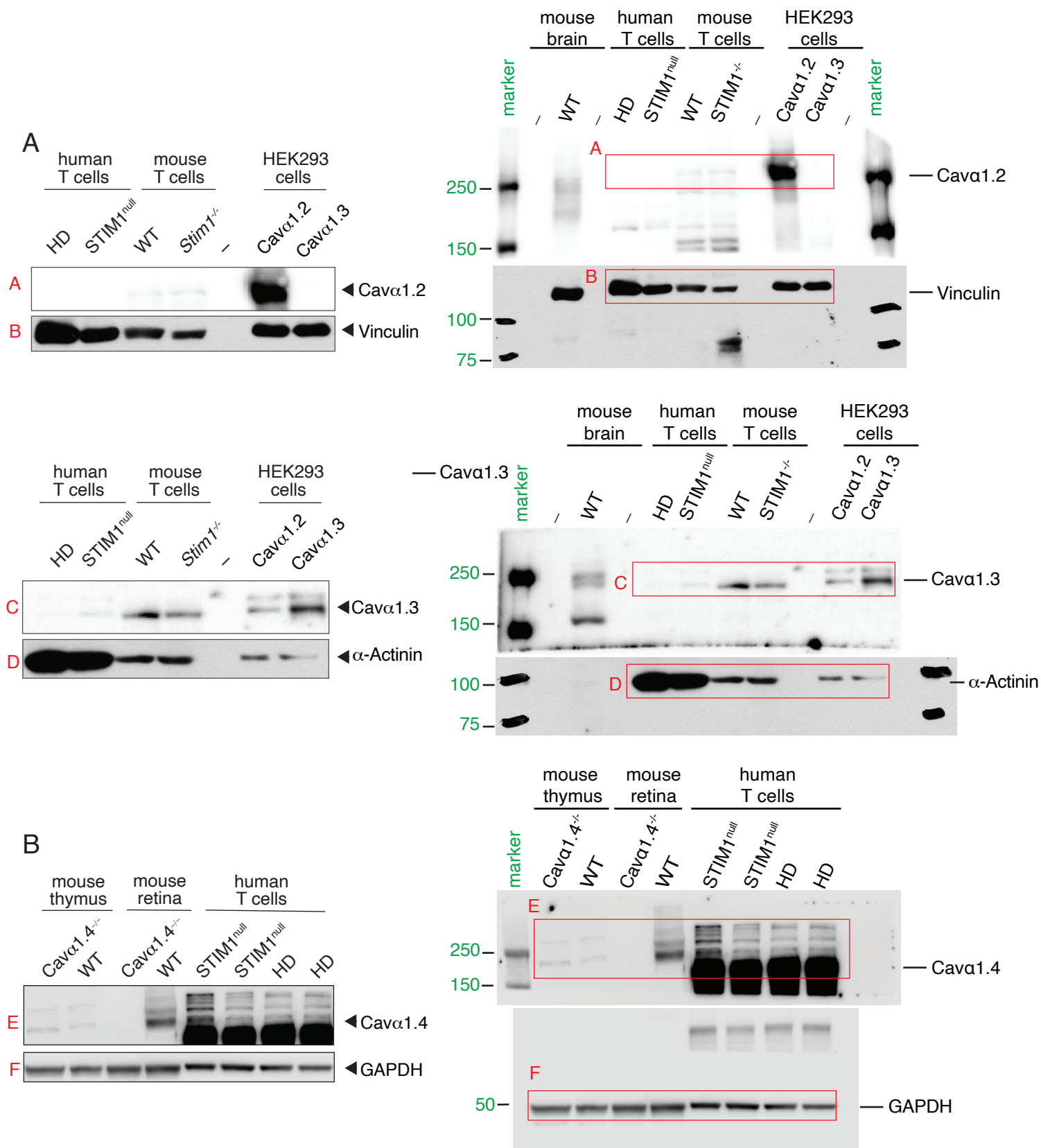

Supplement: Supplementary file 4 — Source Data [file 41467_2022_29725_MOESM4_ESM.zip › Source Data/Supplementary Figure 2 and 9 blots.pdf]
